# Special Issue “Nanomaterials for Biomedical and Biotechnological Applications”

**DOI:** 10.3390/nano12111923

**Published:** 2022-06-04

**Authors:** Angelo Ferraro

**Affiliations:** School of Electrical and Computer Engineering, National Technical University of Athens, Zografou Campus, Zografou, 15772 Athens, Greece; an.ferraro2@gmail.com

In the last few decades, biomedical and biotechnological researchers have turned their interest to nanocomposite materials. One of the first applications of nanomaterials in medical fields was the use of Gadolinium and Iron Oxide nanoparticles as contrast agents in MRI imaging. Another well-established clinical application of nanomaterials is the use of gold nanoparticles to enhance the performance of rapid screen tools such as lateral flow tests, widely in use nowadays for the COVID-19 pandemic. Since these very first applications, a growing body of scientific work has been published showing the extraordinary advantages offered by nanomaterials in speeding up chemical reactions, enhancing the yield of cell-based catalysis, ameliorating the performance of medications and many other health care services.

The need for constant improvement to reach a high standard of safety and to make nanomaterials accessible for marketing has generated, as mentioned before, a considerable number of scientific papers that highlight new important aspects to be considered, such as synthesis, stability, biocompatibility, and easy manipulation. In order to provide a comprehensive update on the latest discoveries concerning the biomedical and biotechnological applications of nanomaterials, this Special Issue of *Nanomaterials* collected 14 papers: 10 as research articles and 4 as reviews.

The topics analysed in this Special Issue focus on the use of magnetic iron oxide nanoparticles, gold nanoparticles functionalized ciprofloxacin, boron nitride nanotubes, poly-ε-caprolactone composite sorbent, titanium nanotubes and chitosan–clay nanocomposites, which have been applied in both biomedical and biotechnological fields. On the other hand, the review papers focus on nanotechnology-based diagnostic and therapeutic approaches to tackle colorectal cancer and SARS-CoV-2-related pathologies; nanomedicine applications for ocular drug delivery and the different methods to synthetize cerium oxide nanoparticles meant to be used as a clinical tool.

Iron Oxide nanoparticles present low cellular toxicity, can be easily synthesised and, most importantly, are easily functionalized with a variety of chemical groups. Four papers have been published showing the versatility of iron oxide nanoparticles. The work of G. Banis et al. [1] focuses on the advantages presented by the superparamagnetic feature of iron oxide nanoparticles, highlighting that they are magnetic only when surrounded by a magnetic field. Such a property allows to remotely control and thus drive magnetic nanoparticles using a proper magnetic field, which can be generated either by permanent magnets or by electromagnets. In their paper, G. Banis and colleagues describe an ingenious electromagnet apparatus with the ability to precisely push small drops of magnetic nanoparticles. The novelty of this research is the possibility to turn, push or attract magnetic nanomaterials by controlling the intensity of the electric current feeding the electromagnet array. The paper is accompanied by a video (available on *Nanomaterials* Journal), where the authors show the path of magnetically driven nanoparticles suspended in mineral oil. The clinical implications of these achievements are several, indeed it is now possible to hypothesise tissue and organ-specific drug administration by conjugating medications to magnetic nanoparticles and subsequently using tuneable magnetic fields to reach the internal target sites.

In addition to superparamagnetism, iron oxide nanocomposites present another interesting physical property: the ability to increase their temperature up to hundreds of degrees Celsius when embedded in an alternating magnetic field; the so-called magnetic hyperthermia. Such controlled thermal energy can be used as a physical tool to specifically kill, e.g., dangerous tumour cells. Sánchez et al. [2] performed an interesting study on how to produce cubic magnetic nanoparticles with sizes lower than the monodomain critical value while maintaining the supermagnetism critical value, which guarantees no agglomeration and, thus, nanoparticles remain in a colloid state when in solution. They optimised several synthesis variables such as ramp-up temperature, stirring speed, type of solvent and proportions of raw materials in order to achieve the production goals. Another important issue examined in the study was the economic and technological viability of production in order to set the path for future industrial implementation to support clinical widespread applications of magnetic hyperthermia against tumours.

In this Special Issue, the use of nanoparticles to tackle cancer malignancies both at diagnostic and at therapeutic levels have also been addressed by an excellent review paper prepared by Al-Joufi and colleagues [3]. The review, in the first part, provides an exhaustive but simple overview of colorectal cancer (CRC) disease and the standard diagnostic tools, whereas in the second part it presents the use of advanced nanomaterials for CRC diagnosis and treatment.

Another important clinical topic treated in the Special Issue is antibiotic resistance towards drugs used to cure infectious diseases caused by a variety of microbes. This phenomenon is becoming a global concern since a growing number of bacteria strains are now resistant to treatments, presenting a significant threat for human health. Nawaz and colleges [4] present an interesting paper on how to enhance the efficacy of the antibiotic Ciprofloxacin by conjugating it to gold nanoparticles. The research focuses on the synthesis and physical characterization of ciprofloxacin-loaded gold nanoparticles (CIP-AuNPs) and their effect on the colonisation of the bacterium Enterococcus faecalis in the liver and kidneys of mice. Authors demonstrate the effectiveness of CIP-AuNPs in eradicating E. faecalis from the host tissues and, unlike CIP alone, CIP-AuNPs were non-haemolytic, thus enhancing also the drug’s safety.

Matters of improving safety and biocompatibility have been tackled also for orthopaedic implants that are used to repair bone traumas and damages. Baker et al. [5] propose in their research paper that a new surface treatment with Titanium nanotubes (TiNT) of implants may promote and improve osseointegration—a phenomenon that leads to the integration of the implant in the damaged bone. In the study, local and systemic responses to aligned and trabecular TiNT (the two most common TiNT surface morphologies) are assessed through in vitro cellular, as well as in vivo, experiments.

In the contest of nanomedicine, Silva et al. [6] report how to improve the performance of boron nitride nanotubes (BNNTs), which are employed in several medical areas. They combined copper-64 with BNNTs and synthesized and characterized 64Cu-BNNTs with appreciable properties that suggest numerous multifunctional applications, with advantages for cancer diagnosis and therapy.

Suresh and colleagues [7] also focus their work on biomedical field and in particular on how to improve drug activity to tackle rheumatoid arthritis. In their research paper it is show a novel formulation of a methotrexate-loaded nanoemulsion for subcutaneous administration. Even though the paper presents initial data that must be confirmed, the authors show that methotrexate-loaded nanoemulsion, at lower concentration, is safer for systemic circulation and that anti-arthritic activity of the nanoemulsion in CFA-induced animals is superior compared to the market drug.In connection with the previous studies, the group of Veloso and colleagues [8] reported the performance improvement of drug carrier-like peptide-based hydrogels by using two different functionalized magnetic particles. The paper showed how to use two different functionalized nanoparticles, citrate-stabilized and lipid-coated magnetic nanoparticles, in the formation of dehydropeptide-based supramolecular magnetogels. These nanocomposites have been tested for the encapsulation and release of Doxorubicin—one of the most commonly used chemotherapeutic drugs in a wide variety of cancers.

An interesting review is also presented by Khiev and colleagues [9], who analysed the topic of nanotechnology-based drug delivery systems in treating ocular diseases. The review highlights the potential of nanomaterial tools to overcome several limitations of conventional therapies during drug delivery across the blood–retinal barrier, making it a major clinical challenge. Therefore, the review summarizes the development of organic and inorganic nanoparticles for ophthalmic applications.

The use of nanomaterials to tackle other human health threats such as the SARS-CoV-2 pandemic is similarly reviewed by Asdaq and colleagues [10] and published in the Special Issue. The review, in the first part, focuses on the virus’s basic structure, pathogenesis, and the current treatment options for COVID-19. In the second part, the study addresses nanotechnology and its applications in diagnosis, prevention, treatment, and targeted vaccine delivery, laying the groundwork for a successful pandemic fight.

Another critical review study is presented by Nyoka et al. [11], where the authors focus on the synthesis of cerium oxide nanoparticles by analysing various methods and explaining how the synthesis method affects physicochemical properties, behaviour in biological environments and catalytic abilities, as well as toxicity.

Concerning the chemistry application of nanomaterials, the theme of complex biological mixture separation is discussed by Raabová and colleagues [12]. In their research paper, the authors describe the advantages of combining poly-ε-caprolactone micro- and nanofibers for the online extraction of analytes from milk and serum matrixes, and confirm the protein removal capability, good mechanical stability and re-usability of this sorbent in more than 300 analyses.

Finally, the following two papers of this Special Issue address biotechnological applications.

M. Savvidou et al. [13] illustrate in their work a biotechnological application of iron oxide nanoparticles. Additionally, in this case they take advantage of the superparamagnetic property of iron oxide nanoparticles. They report how to harvest microalgae cells from the culture medium. Microalgae are photosynthetic single cell organisms that grow both in sea and fresh water. These marvellous forms of life are able to synthetise good quality proteins, lipids, antioxidant, food dye and other metabolites used for food and cosmetic applications. They require water medium to grow and, in order to collect their valuable products, must be separated from their culture liquid. Several de-watering technologies are available, such as filtration, centrifugation, precipitation, decantation, but all of them are time-consuming, high energy-demanding and expensive. M. Savvidou and her group report how the use of magnetic nanoparticles improved de-watering yield and at the same time lowered time and cost of microalgae harvesting.

The work of Benucci et al. [14] focuses on the development of new nanocomposite materials for food application. In their research, the authors synthetised chitosan–clay nanocomposite films to be used as carriers for the covalent immobilisation of the proteolytic enzyme, Papain. This enzyme is used to stabilise various types of food and drinks. The paper demonstrates how the use of chitosan–clay nanocomposite enhances the ability of Papain to stabilise white wine.
